# Instrumental Odour Monitoring System Classification Performance Optimization by Analysis of Different Pattern-Recognition and Feature Extraction Techniques

**DOI:** 10.3390/s21010114

**Published:** 2020-12-27

**Authors:** Tiziano Zarra, Mark Gino K. Galang, Florencio C. Ballesteros, Vincenzo Belgiorno, Vincenzo Naddeo

**Affiliations:** 1 Sanitary Environmental Engineering Division (SEED), Department of Civil Engineering, Università degli Studi di Salerno, Via Giovanni Paolo II, 132, 84084 Fisciano (SA), Italy; v.belgiorno@unisa.it (V.B.); vnaddeo@unisa.it (V.N.); 2 Environmental Engineering Program, University of the Philippines, Diliman, Quezon City 1101, Philippines; mkgalang@up.edu.ph (M.G.K.G.); fcballesteros@up.edu.ph (F.C.B.J.)

**Keywords:** artificial neural network, data extraction, electronic nose, linear discriminant analysis, odour classification monitoring model

## Abstract

Instrumental odour monitoring systems (IOMS) are intelligent electronic sensing tools for which the primary application is the generation of odour metrics that are indicators of odour as perceived by human observers. The quality of the odour sensor signal, the mathematical treatment of the acquired data, and the validation of the correlation of the odour metric are key topics to control in order to ensure a robust and reliable measurement. The research presents and discusses the use of different pattern recognition and feature extraction techniques in the elaboration and effectiveness of the odour classification monitoring model (OCMM). The effect of the rise, intermediate, and peak period from the original response curve, in collaboration with Linear Discriminant Analysis (LDA) and Artificial Neural Networks (ANN) as a pattern recognition algorithm, were investigated. Laboratory analyses were performed with real odour samples collected in a complex industrial plant, using an advanced smart IOMS. The results demonstrate the influence of the choice of method on the quality of the OCMM produced. The peak period in combination with the Artificial Neural Network (ANN) highlighted the best combination on the basis of high classification rates. The paper provides information to develop a solution to optimize the performance of IOMS.

## 1. Introduction

Instrumental odour monitoring systems (IOMS) are devices that function as an artificial paradigm of the olfactory stimuli to reveal environmental odours. Their general architecture is composed of a sampling system, along with a detection unit, in which the array of gas sensors and signal processing system are located [1,2,3,4]. There are wide and different gas sensor technologies currently available [1,3]. In 2015, within the framework of CEN/TC 264—Air Quality, a new working group (WG41) with the aim of proposing a new European standard for IOMS environmental odour monitoring applications was started [5]. IOMS has gained a great deal of popularity and applicability over the last few years in the field of air quality and, in particular, for the monitoring of odours due to the annoyance and impact induced by the growing number of emissions in the environment by industrial activities [5,6]. Furthermore, IOMS possessed numerous advantages over sensorial (e.g., dynamic olfactometer) and analytical instrument (e.g., Gas chromatography–mass spectrometry, colorimetric method, catalytic, infrared and electrochemical sensors, photoionization detector, differential optical absorption spectroscopy) because it is applicable for in-situ and in real time measurements [5,6,7,8]. Meanwhile, other techniques are combined with IOMS, such as pre-concentrator (i.e., silicon-micro), to improve the recognizing capability [9], Bluetooth to a smartphone for remote-controlling applications [4], and GC-MS to identify the responsible gas compounds in odour emission [10,11,12]. IOMS output odour metrics may include odour classification and/or odour quantification [13]. In order to use an IOMS, first, a training phase is needed, which aims to create the odour monitoring model (OMM). Generally, the sequence to obtain the OMM consists of three steps: raw data acquisition; signal processing and dimensionality reduction of the acquired data; and pattern-recognition algorithms applications [6,14]. One of the benefits of the IOMS technology is that it allows for continuous measurements, making it possible to achieve a real time control, which is ideal for environmental odours monitoring [14,15,16,17,18,19]. However, the IOMS accuracy to a specific on-site application requires further improvement and is still being studied. IOMS innovation can be in terms of hardware and/or software development of the system [19]. The hardware modification could be in terms of the selection of sensitive material, optimization of number and typologies of the sensors array, and in the implementation of specific signal control and management unit (span and calibration system) [20]. Meanwhile, the software development can be applied in terms of the feature extraction of the data and assignment of the appropriate pattern recognition algorithm [20,21,22,23]. The internal function directs the IOMS to perform, in an intelligent way, recognizing and interpreting the information in a fast and robust manner [21].

Many studies feed the complete signals as input to the pattern-recognition algorithm, which makes the system computationally expensive, complex, and hard to implement and requires a large memory space [19,22]. Due to large number of values, feature extraction of signals is used to eliminate redundant data and improve the accuracy of the IOMS [24,25,26,27]. By applying this method, the most important data from large set of signals can be captured, resulting in a reduction in computation time, as well as an increase in the speed of measurement and storage [28,29]. Different feature extraction techniques are available in current literature, such as data extraction from the original response curves, from curve fitting parameters, from transform domain, from phase space, etc. [19,30,31] (Table 1).

The above-mentioned techniques have been used in recent years, while new methods are starting to be recognized, such as phase space (PS), dynamic moments (DM), parallel factor analysis (PARAFAC), energy vector (EV), power density spectrum (PSD), surface electromyography (sEMG), windowed time slicing (WTS), etc. [20,21]. In practical applications, the extraction from the original response curves represents one of the most used techniques, due to its intuitive nature and fast calculations [19,20]. Selecting the useful data can improve the discrimination function and exclude values that can cause noise and uncertainty in the measurement. Moreover, to maximize the potential of IOMS, the extracted data and the pattern-recognition technique must work together. Pattern-recognition techniques are mathematical models (i.e., statistical and biological) that are used to establish a relationship between input variables (independent variable) to the target output (dependent variable) in the dataset. The mathematical treatment of correlation of the odour metric with human odour perception is particularly important, and to be stressed in the application for odour monitoring, due to the large number of odourants that cause the odour [13,34]. Table 2 reports an overview of the principal pattern-recognition techniques applied to IOMS technologies.

The research presents and discusses the influence of the application of different extracted signals and pattern recognition methods in the elaboration of the environmental odour classification monitoring model (OCMM) with IOMS. The paper aims to optimize the performance and robustness of an IOMS. The piecemeal signals (i.e., rise, intermediate, and peak state) obtained from the original response curves in combination with the use of the Linear Discriminant Analysis (LDA) or the Artificial Neural Networks (ANN) as pattern recognition techniques are investigated and argued. Laboratory experimental analysis with real samples were considered, to analyze and compare the results.

## 2. Materials and Methods

### 2.1. Experimental Setup

Research studies were carried out by collecting real odour samples at a complex industrial petrochemical plant. Two odour classes were sampled directly at the emission of two different sources in floating roof storage tanks in accordance with the EN 13725 (2003), by using a static lung effect device.

Ten samples Class A (“petrol”, CAS 86290-81-5) and 13 samples Class B (“diesel”, CAS 68476-34-6) were collected, with a weekly frequency, in nalophan bags of 7-L volume. Moreover, 28 ambient air (Class C) samples were collected in the field surrounding the plant, to distinguish among the odours from the investigated sources and the ambient air (no annoyance). A total of 51 samples from three odour classes were used for the research.

### 2.2. IOMS Technology and Data Acquisition

The seedOA IOMS technology, developed by the Sanitary Environmental Engineering Division (SEED) of the University of Salerno, Italy, was used for the experiments. The functional architecture of the seedOA consists of a sampling system, a detection unit, a signal processing system, and a control and management system [41,42,43,44]. The sampling system contains a specific unit that allows the standardization of the temperature and humidity conditions of the analyzed gaseous sample. The air from the sample is drawn by a pump located downstream of the measuring chamber with a constant flow rate of 300 mL m^−1^. The detection system is composed by the code^®^ measurement chamber [44], which contains total of 16 sensors distributed on two different levels. For the specific research, thirteen of the overall installed sensors are of metal-oxide semiconductors type (MOS, Figaro) and adopted for the measurement (Table 3), while the other three sensors are inserted for the control of the environment and process parameters (temperature, humidity and flowrate).

### 2.3. Odour Classification Monitoring Model (OCMM) Elaboration

#### 2.3.1. Data Acquisition

All the collected gas samples were individually acquired by the seedOA IOMS technology adopting an odour‒odourless air cycle [13,34]. An acquisition time and a recovery time of 2 min were set for each sample, with a data detection time step of 2 s. A total of 60 data points for each sample were recorded. The seedOA IOMS measured the resistance of the sensors by a voltage divider. Odourless air was used to recover the base resistance of the sensors each time before the next measurement.

#### 2.3.2. Data Reduction

**Figure 1:**
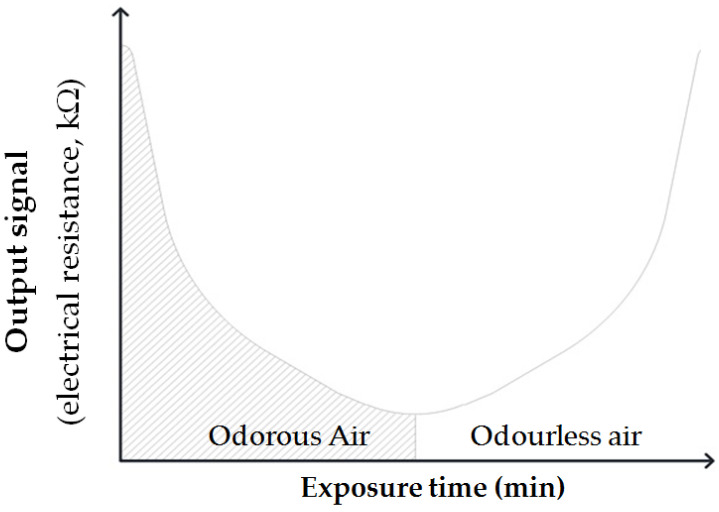
Electrical signals trend with respect to time at odour and odourless air exposure.

The signal responses provided by the sensors are pre-elaborated and given in fractional change in resistance and registered as kΩ (*R_S_ = (R − R_O_)/R_O_*, where *R* is the resistance value after the reaction with a gaseous compound, and *Ro* is the default resistance value of the sensor (baseline resistance)) [20,21,45]. For the MOS sensors, the relationship between resistance and the gas concentration is inversely proportional and of the type:

R (kΩ) = A(C)^–α^, (1)

where: R is the electrical resistance supplied by the sensor; A is a constant defined by the material (e.g., TiO_2_, ZnO, SnO_2_, etc.); C is the concentration of analyzed gas; and α is the slope (e.g., experimental quantity of the gas). Figure 1 reports the general trend of the output signal response provided by the sensors, expressed in terms of electrical resistance (e.g., kΩ) with respect to exposure time (e.g., minute) and presence of odour and odourless events. As shown, when the sensor is exposed to odour, its output signal in terms of resistance decreases, while, when exposed to odourless air, the signal in terms of electrical resistance returns to the initial reference base values.

**Figure 2:**
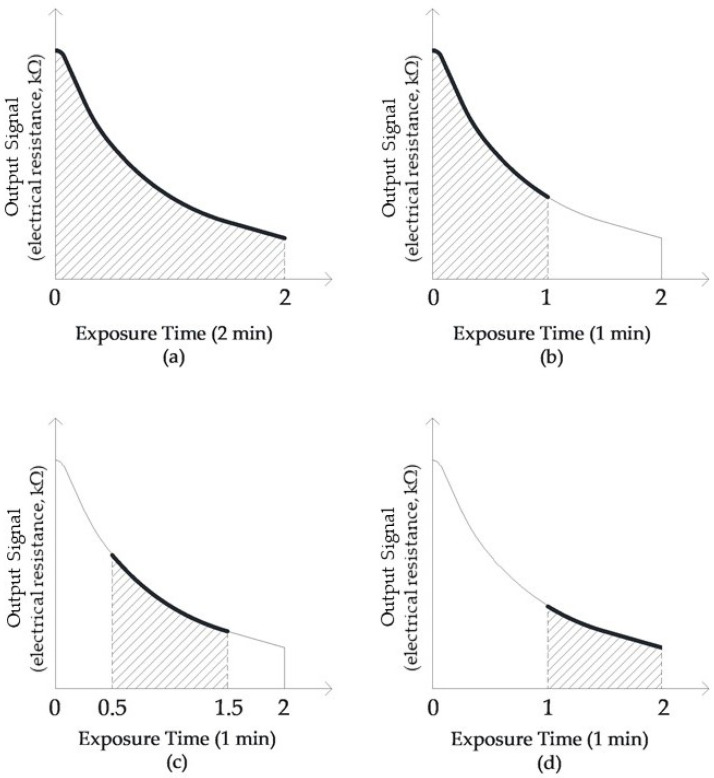
Extracted signals at different points (**a**) complete sensors response curve; (**b**) rise period; (**c**) intermediate period; (**d**) peak period.

For the experimental activities, in addition to considering the data of the complete sensors response curve (i.e., Figure 2a), piecemeal signals lasting 1 min, such as the rise period (first 1 min of acquisition, Figure 2b), the intermediate period (intermediate, 1 min of acquisition, after 30 s from the start, Figure 2c), and the peak period (last 1 min of acquisition, Figure 2d), during the 2-min acquisition time, were extracted and investigated.

#### 2.3.3. Pattern-Recognition Algorithms

Linear discriminant analysis (LDA), as a traditional statistical method, and artificial neural network (ANN), as a biological method, were used to investigate the influence and effect of the application of different categories of pattern recognition algorithms.

Linear discriminant analysis (LDA) adopts linear combinations of variables to distinguish between classes that results in linear decision boundaries. The method searches for a linear transformation that maximizes class separability in a reduced dimensional space [32,38,46]. LDA is a popular classifier technique and commonly used in IOMS technologies for environmental odour monitoring and assessment [34,35]. During LDA training, coefficients (i.e., *k, a, b … α*) of different discriminant function (*γ*) equations per representative group (i.e., *λ, β* and *ω*) are calculated. In predicting the categories of the new data, the input values are substituted to the variables (i.e., *x_1_, x_2_ … x_n_*) of the equations reported below (Equation (2)) to measure the scores:

γ_λ_ = *k*_1_ + *ax*_1_ + *bx*_2_ + … *αx_n_*, (2)

γ_β_ = *k*_2_ + *ax*_1_ + *bx*_2_ + … *αx_n_*, (3)

γ_ω_ = *k*_3_ + *ax*_1_ + *bx*_2_ + … *αx_n_*. (4)

The highest score indicates the group where those values belong.

**Figure 3:**
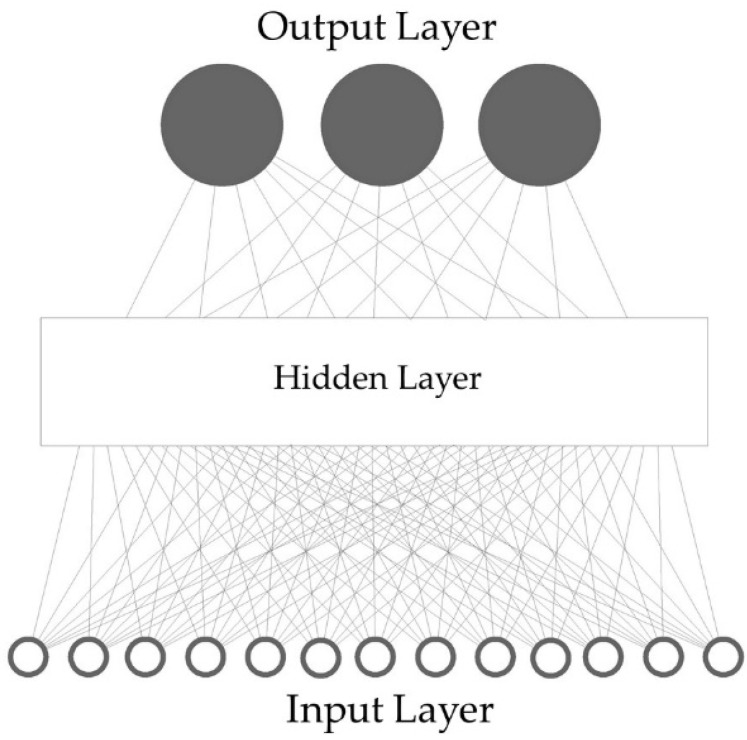
Structure and topology of the artificial neural network pattern-recognition algorithms.

Meanwhile, artificial neural network (ANN) is biological paradigm that serves as mathematical models in simulating complex systems and considered black-box [47,48,49,50,51,52]. A general ANN consists of input neurons, hidden neurons, and output neurons, connected via synapse, which contains specific weight values [49,50]. For the experimental activities, a 3-layer feed-forward neural network was designed. The 13 different electrical resistance profiles from seedOA IOMS were used as input data, while the three investigated odour classes were used as target output (Figure 3). The ideal number of neurons is identified by means of “trial-and-error” on the basis of high correlation values (R^2^) and classification rates (%) between measured and predicted output.

In training the neural network, the system optimizes the ideal weight values until the loss function is minimized under the influence of a learning algorithm [49,50]. The Bayesian Regularization algorithm was applied, introducing a non-linearity by using a tan-sigmoid function to reduce the possibility of an over-fit since it uses a probabilistic nature for the network weights [53].

#### 2.3.4. Training and Validation datasets

The overall acquired dataset in terms of fractional change in resistance, for each of the 13 odour measurement sensors, at a given time of the overall sample acquisition (one data every 2 s), was divided into a “training” and a “validation” dataset. The training dataset was used to determine the coefficients of the two investigated mathematical models, considered subsequently for the validation stage. The validation dataset, consisting of six separate sets of samples and applied according to “leave-one-group-out” method [24], was adopted as test samples to verify the model accuracies.

For the LDA training, the datasets were organized and labeled according to the three investigated odour classes (G1 = Class A, G2 = Class B, and G3 = Class C) (Table 4).

For the training datasets, by applying the ANN, supervised learning was adopted. Binary classifiers, such as “1” and “0”, were assigned in the output to group the data, where “1” refers to the belonging to the group, while “0” indicates no interaction (Table 5).

To assess the reliability of the trained models, a validation test was conducted, using the overall acquired data, the data excluded from the training dataset, and considering known the source for the comparison test (Table 6). The accuracy rates are, therefore, defined by analyzing the known values with the predicted ones.

### 2.4. Statistical Analysis

Excel 2010 software (Microsoft, Washington, DC, USA) was applied for the pre-processing data extraction. Meanwhile, Statistica 10 (StatSoft, Tulsa, OK, USA) and MATLAB R2017a (MathWorks, Natick, MA, USA) were used as the computational software for the LDA and ANN pattern-recognition algorithms elaboration, respectively.

For the LDA applications, a scatterplot diagram and confusion matrix was used to analyze the behavior of the detected data points per investigated odour classes and to evaluate the performance of the predicted classification algorithm.

For the ANN methodology, the coefficient of determination (R^2^) and the mean square error (MSE) were calculated to investigate the relation of the predicted and measured data, as well as to update the weights of the number of times all of the training vectors.

### 2.5. Comparison Studies

Comparative analyses of the different Odour Classification Monitoring Models (OCMMs), elaborated by using the different future extraction techniques and pattern recognition methods, were performed by calculating the classification accuracy rate during the training and validation tests, per investigated odour class (*α_i_; i* = class) and for all the detected data (*φ*):

(5) $$\alpha_{i}\left(\%\right)=\frac{number\;of\;correctly\;classified\;data}{total\;number\;of\;detected\;data}\;\times \;100\;\varphi \;\left(\%\right)=\frac{1}{ɳ\;}\overset{3}{\underset{i=1}{\sum }}\left(\alpha_{i}\right),$$

where *α*% is the individual accuracy rate per class, and *φ%* is the overall accuracy rates (i.e., summation of the individual accuracy rates (α%) divided by the total number of class (*η*)).

A total of eight (8) OCMMs were elaborated and compared (Table 7).

## 3. Results

### 3.1. OCMMs Using Different Extracted Signals and LDA Application

Table 8 summarizes the results of the classification accuracy rate obtained by applying the LDA model and the training dataset. Each column shows the classification rate per investigated piecemeal signal. The values of the Wilks’ lambda are also highlighted to analyze the degree of the discriminatory power of the model.

Among the different feature extractions, the peak and the intermediate period show the highest level of confidence in discriminating all the analyzed data in the training phase.

For all the investigated features, despite the adjustment made to some parameters during the training (i.e., the tolerance value), the Wilks’ lambda values are near to 0, thus demonstrating general good discrimination properties for all three classes.

The results clearly highlight an influence in the classification accuracy rate determination, in relation to the choice of the piecemeal signal. Considering the analysis per investigated odour class, a maximum variation of 21.61% of the classification accuracy rate was detected for Class A by using, respectively, the “peak” or the “rise” signal, whereas a minimum variation of 0.23% was observed for the “Ambient Air” odours class (Class C).

While performing the analysis with all the detected data (φ), the discrimination variation of the investigated samples by adopting different extracted signal is equal to 4.49%.

**Figure 4:**
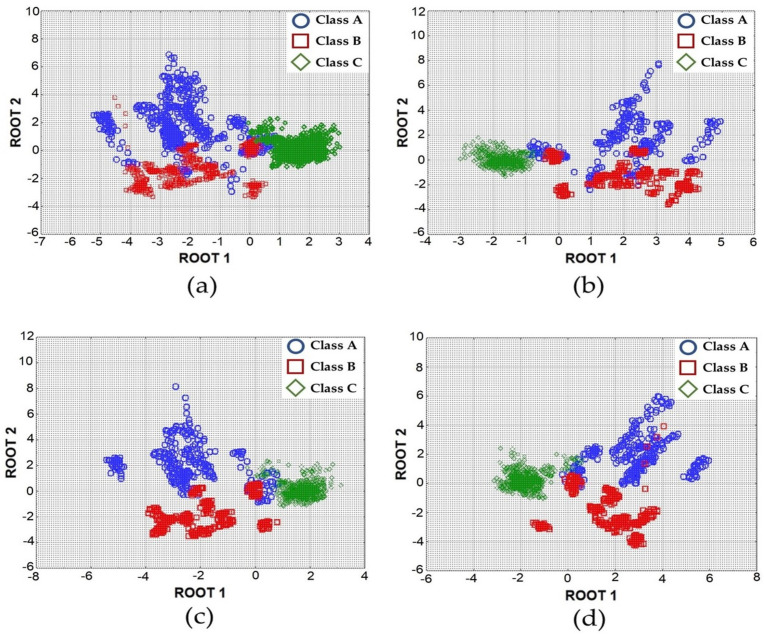
Scatterplots for the first two roots at different data extraction ((**a**) complete response curve; (**b**) rise period; (**c**) intermediate period; (**d**) peak period).

Figure 4 shows the scatter plots produced from the linear discriminant analysis (LDA) of the “training” dataset, with all the detected data, showing a distinction among Class A, Class B, and Class C by using the (a) complete response curve data and extracted data for the (b) rise data, (c) intermediate data, and (d) peak data.

The results also graphically confirm that the peak analysis (Figure 4d) shows better cluster formation of the classes, and Class C (ambient air) is the most recognizable class among the different investigated classes. A more pronounced difficulty of discrimination is shown, especially, among some elements of the classes A and B for all of the investigated piecemeal signal features. The cause may be related to the relatively small magnitude and difference in resistance values detected by the sensors solicited and, probably, to the similar composition in terms of predominant odourous substances and/or odour concentration of the investigated samples.

Table 9 summarizes the classification metrics during the LDA validation test, determined by using the discriminant factors equation developed in the training phase.

Excellent classification accuracy rate results are highlighted only for the Class B samples, while a much lower recognition percentage was detected for the Class A samples. No samples of the Class C were correctly identified. Once again, the analyses show the better response by using the peak or the intermediate data.

### 3.2. OCMMs Using Different Extracted Signals and ANN Application

MATLAB environment has a default setting that automatically partitions the input dataset into 70–30% (i.e., train-test set) during training. The purpose of this configuration is to eliminate the possibility of over-fitting. Each piecemeal signal was tested at different number of neurons in the hidden layer. The ideal ANN topology was found at “13-7-3”. Table 10 summarizes the coefficient of determination (R^2^) obtained by applying the ANN during the training stage.

Considering the overall R^2^ to assess the ANN accuracy, the results show that all the correlations (R^2^) were found to be >0.998 for all the subsets of the extracted signals. This means that the ANN was able to detect all the possible interactions in the dataset.

**Figure 5:**
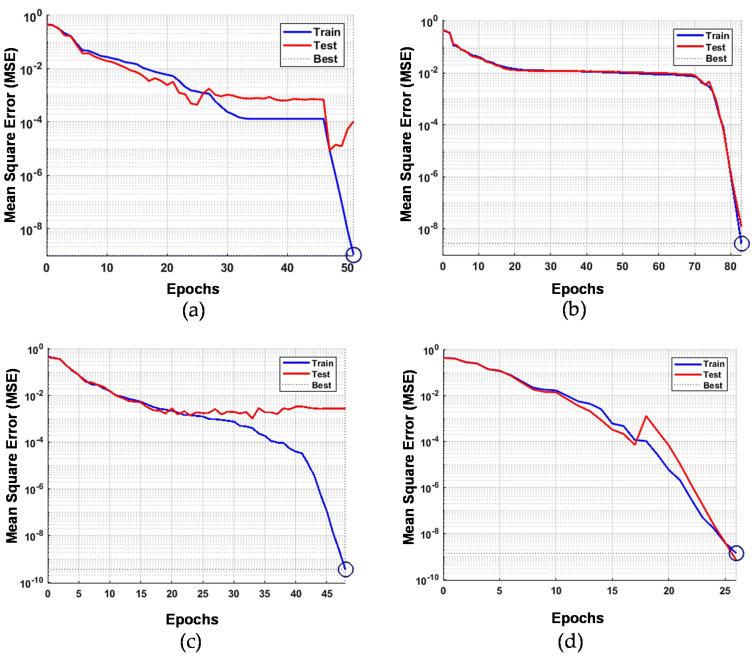
ANN performance at investigated extracted signal ((**a**) complete response curve; (**b**) rise period; (**c**) intermediate period; (**d**) peak period) with respects to mean square error versus the number of epochs.

Figure 5 highlights the graphical representation of the results summarized in Table 10 to evaluate the R^2^ trend through the mean square error (MSE) vis-a-vis the number of epochs, by using the different sets of extracted data.

The ANN was able to map good patterns, especially when the data in the rise and peak part are utilized in the basis of small MSE at low number of epochs. The best training performance was found, respectively, to be 1.00 × 10^−9^ at epoch 51 for the complete response curve data (Figure 5a), 2.60 × 10^−9^ at epoch 83 for the rise part data (Figure 5b), 3.49 × 10^−10^ at epoch 48 for the intermediate data (Figure 5c), and 1.38 × 10^−9^ at epoch 26 for the peak data (Figure 5d).

Table 11 summarizes the classification metrics during the ANN validation test, determined using the values of the weights and biases, encoded as coefficients to satisfy the topology of “13-7-3” generated during the training.

The results show that the ANN misclassified Class C (ambient air) data. However, a perfect classification (100%) was achieved for Class A and Class B using the intermediate and peak data points. This scenario might be attributed to the idea that molecules of Class A and Class B are more sensitive to the gas sensors, in which an observable reaction is recognized when compared to Class C. The highest overall recorded accuracy was determined equal to 66.67% for the intermediate and peak data points.

### 3.3. Comparison Studies

Table 12 presents and compares the classification accuracy rates obtained in the training and validation stage through the application of the LDA and ANN, along with the different extracted signal points, by performing the analysis with all the detected data (*φ*).

The peak steady part is confirmed as the piecemeal signal that provides the highest discriminatory value for all the investigated cases and contains the most useful information for both the pattern-recognition techniques. Despite the complete response signals containing the complete information, this condition appears to slow down the performance of the pattern-recognition algorithm. A good classification accuracy was highlighted by using the intermediate period of the overall acquired data.

In the LDA classification, the technique was able to discriminate groups with a good satisfaction rate (>89.71%); however, when simulated with unknown data during validation, the model could not classify them higher than 50%. This phenomenon might due to the natural characteristic of the technique in relying on normal data distribution. However, some variables do not obey this behavior. Meanwhile, by applying the ANN technique, the results are relatively higher than in LDA. The model acquired a high learning condition, which is manifested by the classification rates for all the piecemeal signals and principally by using the intermediate and peak signals during the validation stage. The ANN demonstrates a better pattern-recognition potential than using the LDA for almost all the experiments carried out (e.g., +8.22% and even +12.71% during the training phase, considering, respectively, the peak or rise periods). The cause may be related to the higher ability of the ANN technique to deal with the noise in the dataset. This characteristic is an asset of the ANN due to the fact that gas movements are dynamic. Only during the validation phase by using the rise signal, LDA highlights a better classification accuracy.

## 4. Conclusions

The analysis of the adoption of different fragmented signals from the overall acquired data and their responses with different pattern-recognition algorithms, such as LDA and ANN in the OCMMs elaboration with IOMS, highlight the influences in the final classification accuracy. For the investigated analyses, during the LDA training, the intermediate and peak periods had the highest discrimination rates. On the other hand, during the ANN training, all the fragmented signals performed well in terms of a high R^2^, low MSE, and high classification metrics. ANN proves to have a higher learning capability than LDA, while, during the test set validation of the two models, the intermediate and peak parts confirm the highest accuracy, and ANN outperforms LDA in almost all the investigated cases.

The selection of the feature extraction can optimize the IOMS performance by capturing the most important signals to improve the system suffering from a large dataset and memory storage space. In this way, the redundant signals that may contribute to the uncertainty in the measurement can be eliminated and increase the robustness of the odour monitoring model. Furthermore, the selection of the most appropriate pattern-recognition technique can improve the overall algorithm of the IOMS, which is manifested by the odour classification metrics.

In LDA, no matter how the parameters were adjusted, such as by lowering the tolerance value, the Wilks’ lambda remains steady, unlike in ANN, where more configurations are still available to explore. Based also on the signal response, the intermediate and peak periods carried the most useful information that can be applied in odour monitoring.

The research can be a guideline for further research on selecting the proper combination of extracted signals and pattern-recognition algorithm. The paper provides useful information for the selection of the most appropriate mathematical data treatment techniques in environmental odour monitoring with IOMS, as well as to promote the development of more flexible systems, in order to minimize redundancy, as well as increase the overall quality and reliability of the system.

## Figures and Tables

**Table 1:** Overview of the principal feature extraction methods and related features.

Technique	Sample Method	Characteristic/s	Equation/s	Reference/s
Extraction from original response curves of sensors	- Steady state models i.e., maximum values (based on electrical resistance)	- the fastest and frequently-used method - some signals are omitted - not applicable for demanding tasks	Difference: *x_ij_* = *R_S_ − R_O_* Relative: *x_ij_* = *R_S_/R_O_* Fractional: *x_ij_* = (*R_S_ − R_O_*)/*R_O_* Logarithm: *x_ij_* = ln (*R_S_/R_O_)* Normalized: *x_ij_* = *x_ij_/*(*R_S_ − R_O_*)	[19,26,27]
Extraction from curve fitting parameters	- Polynomial functions - Exponential functions - Fractional functions	- approximate discrete data using analytical expressions - nonlinear in nature - the fitting process is complicated and long	Polynomial: *y = A_0_ + A_1_^x^ + A_2_^x^ + A_3_x^3^ + …+ A_n_x^n^* $\mathrm{Exponential}:\;y=A_{0}+\sum_{1}^{n}A_{i}+exp(-\frac{x}{T}),$ *i* = *1*,*2*,*3* … Fractional: *y = x/Ax + B*	[28,32]
Transform domains	- Fourier transform - Wavelet transform	- maps the original data into new space - the basis functions are sine and cosine	$\mathrm{Fourier}:\;F(k)=\;\int_{-\infty }^{+\infty }e^{-2\pi ikt}\;xt$*dt* Wavelet (mother): *y_a, b_ =*$\frac{1}{\sqrt{a}}\;$*y*($\frac{t-b}{a})$; *a > 0, -∞ < b < ∞*	[20,33]

**Table 2:** Overview of the principal pattern-recognition techniques used in instrumental odour monitoring systems (IOMS).

Technique	Characteristic/s	Equation	Reference/s
Artificial Neural Network (ANN)	- connect input to output via hidden layers - strong non-linear relationship - independent to assumptions	$y=\sum (i_{1}w_{1}+i_{2}w_{2}+\ldots +i_{n}w_{n}$)	[35,36]
Partial Least Square (PLS)	- connect input to output via latent variables - present multicollinearity - can reduce dimension	*y* = *β_0_* + *β*_1_ *x*_1_ + *β*_2_ *x*_2_ … *β*_n_ *x*_n_ + C	[37]
Linear Discriminant Analysis (LDA)	- combine linear features - locate axes that maximize the variance - can reduce dimension	*y = k_1_+ax_1_+bx_2_…αx_n_*	[32,38]
Multivariate adaptive regression splines (MARSPline)	- a stepwise linear regression - acquire intrinsic complex data map - nonlinear in nature	*y = f (X) = β_0_ +* $\sum_{m-1}^{M}\beta_{m}h_{m}$ *(X)*	[39]
Response Surface Regression (RSR)	- sensitive to dependent variables - good optimization technique - complex configuration	*y* = *β_0_* + *β*_1_ *w* + *β*_2_ *w^2^ + β3 x + β4 x2+ β5 z + β6 z2 + β7 w x + β8 w z + β9 z z*	[40]

**Table 3:** Array of metal-oxide semiconductor gas sensors (MOS) present in the IOMS (seedOA).

Sensor ID	Number	Target Gas
TGS880	2	Alcohols, water vapors
TGS822	2	Alcohols, organic solvent vapors
TGS842	2	Methane
TGS2611	2	Methane
TGS2620	2	Solvent vapors
TGS2602	1	Air contaminants
TGS825	1	Hydrogen sulfide
TGS826	1	Ammonia

**Table 4:** Size of the training dataset for each of the 13 measurement sensors, at different extracted signals, by using linear discriminant analysis (LDA).

Description	Number of Data				Output
	Complete Response Curve	Rise	Intermediate	Peak	Group
Class A	600	300	300	300	G1
Class B	780	390	390	390	G2
Class C	1680	840	840	840	G3

**Table 5:** Size of the training dataset for each of the 13 measurement sensors, at different extracted signals, by using an Artificial Neural Network (ANN).

Group	Number of Data						Assigned Output		
	Complete Response Curve			Rise, Intermediate and Peak					
	A	B	C	A	B	C	A	B	C
Class A	600	0	0	300	0	0	1	0	0
Class B	0	780	0	0	390	0	0	1	0
Class C	0	0	1680	0	0	840	0	0	1

**Table 6:** Size of the validation dataset for each of the 13 measurement sensors, at different extracted signals.

Group	Number of Data						Designated Output		
	Complete Response Curve			Rise, Intermediate and Peak					
	G1	G2	G3	G1	G2	G3	G1	G2	G3
Class A	120	0	0	60	0	0	1	0	0
Class B	0	120	0	0	60	0	0	1	0
Class C	0	0	120	0	0	60	0	0	1

**Table 7:** Matrix of the different elaborated and compared odour classification monitoring models (OCMMs).

Extracted Piecemeal Signal Features	Pattern Recognition Methods	
	LDA	ANN
Complete response curve	OCMM1.1	OCMM2.1
Rise	OCMM1.2	OCMM2.2
Intermediate	OCMM1.3	OCMM2.3
Peak	OCMM1.4	OCMM2.4

**Table 8:** Classification accuracy rate results with LDA application and training dataset.

Group	Classification Accuracy Rate (%)			
	Complete Response Curve	Rise	Intermediate	Peak
Class A	74.83	65.16	80.97	86.77
Class B	79.10	76.92	79.65	78.41
Class C	99.94	100.00	99.88	99.77
OVERALL	89.71	87.29	91.02	91.78
Wilks’ Lambda	0.1091	0.1253	0.0824	0.0714

**Table 9:** Classification accuracy rate results with LDA application and validation dataset.

Group	Classification Accuracy Rate (%)			
	Complete Response Curve	Rise	Intermediate	Peak
Class A	00.00	48.33	50.00	50.00
Class B	98.33	100.00	100.00	100.00
Class C	00.00	00.00	00.00	00.00
OVERALL	32.78	49.44	50.00	50.00

**Table 10:** Correlation values with ANN application during training phase.

Experiment Stage	Complete Response Curve	Rise	Intermediate	Peak
Train (R^2^)	0.9999	0.9999	0.9999	0.9999
Testing (R^2^)	0.9954	0.9999	0.9876	0.9999
Overall (R^2^)	0.9999	0.9999	0.9981	0.9999
Classification Rates (%)	99%	100%	99%	100%

**Table 11:** Classification accuracy rate results with ANN application and validation dataset.

Group	Classification Accuracy Rate (%)			
	Complete Response Curve	Rise	Intermediate	Peak
Class A	76.67	50.00	100.00	100.00
Class B	100.00	50.00	100.00	100.00
Class C	00.00	00.00	00.00	00.00
OVERALL	58.89	33.33	66.67	66.67

**Table 12:** Classification accuracy rates of all investigated conditions.

Extracted Piecemeal Signal Features	*φ_%_* by LDA		*φ_%_* by ANN	
	Training	Validation	Training	Validation
Complete response curve	89.71%	32.78%	99.00%	58.89%
Rise	87.29%	49.44%	100.00%	33.33%
Intermediate	91.02%	50.00%	99.00%	66.67%
Peak	91.78%	50.00%	100.00%	66.67%

## Data Availability

Data available on request due to restrictions.

## References

[1] Giuliani S., Zarra T., Nicolas J., Naddeo V., Belgiorno V., Romain A.C. (2012). An alternative approach of the E-Nose Training Phase in odour Impact Assessment. Chem. Eng. Trans..

[2] Capelli L., Sironi S., Centola P., Del Rosso R., Il Grande M. (2008). Electronic noses for the continuous monitoring of odours from a wastewater treatment plant at specific receptors: Focus on training methods. Sens. Actuators B Chem..

[3] Brattoli M., De Gennaro G., De Pinto V., Demarinis Loiotile A., Lovascio S., Penza M. (2011). Odour Detection Methods: Olfactory and Chemical Sensors. Sensors.

[4] Arroyo P., Melendez F., Suares J.I., Herrero J.L., Rodriguez S., Lozano J. (2020). Electronic Nose with digitl gas sensors connected via Bluetooth to a Smartphone for Air Quality Measurements. Sensors.

[5] Brancher M., David Griffiths K., Franco D., Melo Lisboa H. (2017). A review of odour impact criteria in selected countries around the world. Chemosphere.

[6] Zarra T., Galang M.G., Ballesteros F., Naddeo V., Belgiorno V. (2019). Environmental odour management by artificial neural network—A review. Environ. Int..

[7] Garbacz M., Malec A., Duda-Saternus S., Suchorab Z., Guz L., Lagod G. (2020). Methods for Early Detection of Microbial Infestation of Buildings Based on Gas Sensors Technologies. Chemosensors.

[8] Szulczynski B., Arminski K., Namiesnik J., Gebicki J. (2018). Determination of Odour Interactions in Gaseous Mixtures Using Electronic Nose Methods with Artificial Neural Networks. Sensors.

[9] Slimani S., Bultel E., Cubizolle T., Herrier C., Rouselle T., Livache T. (2020). Opto-Electronic Nose Coupled to a Silicon Micro Pre-Concentrator Device for Selective Sensing of Flavored Waters. Chemosensors.

[10] Gebicki J., Szulczyński B. (2018). Discrimination of selected fungi species based on their odour profile using prototypes of electronic nose instruments. Measurement.

[11] Cui S., Ling P., Zhu H., Keener H.M. (2018). Plant Pest Detection Using an Artificial Nose System: A Review. Sensors.

[12] Marek G., Dobrzanski B., Oniszczuk T., Combrzynski M., Cwikla D., Rusinek R. (2020). Detection and Differentiation of Volatile Compound Profiles in Roasted Coffee Arabica Beans from Different Countries Using an Electronic Nose and GC-MS. Sensors.

[13] Zarra T., Naddeo V., Belgiorno V., Reiser M., Kranert M. (2009). Instrumental characterization of odour: A combination of olfactory and analytical methods. Water Sci. Technol..

[14] Fu J., Li G., Qin Y., Freeman W.J. (2007). A pattern recognition method for electronic noses based on an olfactory neural network. Sens. Actuators B Chem..

[15] Orzi V., Riva C., Scaglia B., D’Imporzano G., Tambone F., Adani F. (2018). Anaerobic digestion coupled with digestate injection reduced odour emissions from soil during manure distribution. Sci. Total Environ..

[16] Ragothaman A., Anderson W.A. (2017). Air Quality Impacts of Petroleum Refining and Petrochemical Industries. Environments.

[17] Kim J.H., Mirzaei A., Kim H.W., Kim H.J., Vuong P.Q., Kim S.S. (2019). A Novel X-Ray Radiation Sensor Based on Networked SnO_2_ Nanowires. Appl. Sci..

[18] Szczurek A., Maciejewska M. (2005). Relationship between odour intensity assessed by human assessor and TGS sensor array response. Sens. Actuators B Chem..

[19] Liu H., Li Q., Yan B., Zhang L., Gu Y. (2019). Bionic Electronic Nose Based on MOS Sensors Array and Machine Learning Algorithms Used for Wine Properties Detection. Sensors.

[20] Yan J., Guo X., Duan S., Jia P., Wang L., Peng C., Zhang S. (2015). Electronic Nose Feature Extraction Methods: A Review. Sensors.

[21] Distante C., Leo M., Siciliano P., Persaud K.C. (2002). On the study of feature extraction methods for an electronic nose. Sens. Actuators B Chem..

[22] Carmel L., Levy S.L., Lancet D., Harel D. (2003). A feature extraction method for chemical sensors in electronic noses. Sens. Actuators B.

[23] Zhang S., Xie C., Zeng D., Zhang Q., Li H., Bi Z. (2007). A feature extraction method and a sampling system for fast recognition of flammable liquids with a portable E-nose. Sens. Actuators B Chem..

[24] Borowik P., Adamowicz L., Tarakowski R., Siwek K., Grzywacz T. (2020). Odor Detection using an E-Nose with a Reduced Sensor Array. Sensors.

[25] Gardner J.W., Boilot P., Hines E.L. (2005). Enhancing electronic nose performance by sensor selection using a new integer-based genetic algorithm approach. Sens. Actuators B Chem..

[26] Sun Y., Wang J., Cheng S. (2017). Discrimination among tea plants either with different invasive severities or different invasive times using MOS electronic nose combined with a new feature extraction method. Comput. Electron. Agric..

[27] Zhang C., Wang W., Pan Y. (2020). Enhancing Electronic Nose Performance by Feature Selection using an Improved Grey Wolf Optimization Based Algorithm. Sensors.

[28] Haddad R., Carmel L., Harel D. (2007). A feature extraction algorithm for multi-peak signals in electronic nose. Sens. Actuators B Chem..

[29] Zhang S., Xie C., Hu M., Li H., Bai Z., Zend D. (2008). An entire feature extraction method of metal oxide gas sensors. Sens. Actuators B Chem..

[30] Liu T., Zhang W., Ye L., Ueland M., Forbes S.L., Su S.W. (2019). A novel multi-odour identification by electronic nose using non-parametric modelling-based feature extraction and time-series classification. Sens. Actuators B Chem..

[31] Yan J., Tian F., He Q., Shen Y., Xu S., Feng J., Chaibou K. (2012). Feature Extraction from Sensor Data for Detection of Wound Pathogen Based on Electronic Nose. Sens. Mater..

[32] Yang W., Wu H. (2014). Regularized complete linear discriminant analysis. Neurocomputing.

[33] Rhif M., Abbes A.B., Farah I.R., Martinez B., Sang Y. (2019). Wavelet Transform Application for/in Non-Stationary Time-Series Analysis: A Review. Appl. Sci..

[34] Zarra T., Giuliani S., Naddeo V., Belgiorno V. (2012). Control of odour emission in wastewater treatment plants by direct and undirected measurement of odour emission capacity. Water Sci. Technol..

[35] Vanarse A., Espinosa-Ramos J.I., Osseiran A., Rassau A., Kasabov N. (2020). Application of a Brain-Inspired Spiking Neural Network Architecture to Odor Data Classification. Sensors.

[36] Wei H., Gu Y. (2020). A Machine Learning Method for the Detection of Brown Core in the Chinese Pear Variety Huangguan Using a MOS-Based E-Nose. Sensors.

[37] Dong Y., Joe Qin S. (2018). Regression on dynamic PLS structures for supervised learning of dynamic data. J. Process Control.

[38] Avila R., Horn B., Moriarty E., Hodson R., Moltchanova E. (2018). Evaluating statistical model performance in water quality prediction. J. Environ. Manag..

[39] Kuter S., Akyurek Z., Weber G. (2018). Retrieval of fractional snow-covered area from MODIS data by multivariate adaptive regression splines. Remote Sens. Environ..

[40] Bezerra M.A., Santelli R.E., Oliveira E.P., Villar L.S., Escaleira L.A. (2008). Response surface methodology (RSM) as a tool for optimization in analytical chemistry. Talanta.

[41] Zarra T., Naddeo V., Belgiorno V. (2009). A novel tool for estimating the odour emissions of composting plants in air pollution management. Glob. Nest J..

[42] Giuliani S., Zarra T., Naddeo V., Belgiorno V. (2013). Measurement of odour capacity in wastewater treatment plants by multisensor array system. Environ. Eng. Manag. J..

[43] Zarra T., Reiser M., Naddeo V., Belgiorno V., Kranert M. (2014). Odour emissions characterization from wastewater treatment plants by different measurement methods. Chem. Eng. Trans..

[44] Viccione G., Zarra T., Giuliano S., Naddeo V., Belgiorno V. (2012). Performance Study of E-Nose Measurement Chamber for Environmental Odour Monitoring. Chem. Eng. Trans..

[45] Jiang W., Gao D. (2020). Five typical stenches detection using an Electronic Nose. Sensors.

[46] Le K.T., Chaux C., Richard F.J.P., Guedj E. (2020). An adapted linear discriminant analysis with variable selection for the classification in high-dimension, and an application to medical data. Comput. Stat. Data Anal..

[47] Galang M.K.G., Zarra T., Naddeo V., Belgiorno V., Ballesteros F. (2018). Artificial neural network in the measurement of environmental odours by e-nose. Chem. Eng. Trans..

[48] Sarigiannis D.A., Karakitsios S.P., Gotti A., Papaloukas C.L., Kassomenos P.A., Pilidis G.A. (2009). Bayesian Algorithm Implementation in a Real Time Exposure Assessment Model on Benzene with Calculation of Associated Cancer Risks. Sensors.

[49] Mjalli F.S., Al-Ashed S., Alfadala H.E. (2007). Use of artificial neural network black-box modeling for the prediction of wastewater treatment plants performance. J. Environ. Manag..

[50] Dharwal R., Kaur L. (2016). Applications of Artificial Neural Networks: A Review. Indian J. Sci. Technol..

[51] Gardner J.W., Hines E.L., Wilkinson M. (1990). Application of artificial neurl networks to an electronic olfactory system. Meas. Sci. Technol..

[52] Jiang Q., Shen Y., Li H., Xu F. (2018). New Fault Recognition Method for Rotary Machinery Based on Information Entropy and a Probabilistic Neural Network. Sensors.

[53] Srivastava N., Hinton G., Krizhevsky A., Sutskever I., Salakhutdinov R. (2014). Dropout: A Simple way to prevent neural networks from overfitting. J. Mach. Learn. Res..

